# High mixed venous oxygen saturation levels do not exclude fluid responsiveness in critically ill septic patients

**DOI:** 10.1186/10326

**Published:** 2011-07-26

**Authors:** Dimitrios Velissaris, Charalampos Pierrakos, Sabino Scolletta, Daniel De Backer, Jean Louis Vincent

**Affiliations:** 1 Department of Intensive Care, Erasme Hospital, Université libre de Bruxelles, Route de Lennik 808, 1070 Brussels, Belgium

## Abstract

**Introduction:**

The aim of this study was to determine whether the degree of fluid responsiveness in critically ill septic patients is related to baseline mixed venous oxygen saturation (SvO_2_) levels. We also sought to define whether fluid responsiveness would be less likely in the presence of a high SvO_2 _(>70%).

**Methods:**

This observational study was conducted in a 32-bed university hospital medicosurgical ICU. The hemodynamic response to a fluid challenge was evaluated in 65 critically ill patients with severe sepsis. Patients were divided into two groups (responders and nonresponders) according to their cardiac index (CI) response to the challenge (>10% or <10%).

**Results:**

Of the 65 patients, 34 (52%) were fluid responders. Baseline SvO_2_, CI, heart rate (HR) and mean arterial pressure (MAP) were not statistically different between groups. The responders had lower pulmonary artery occlusion pressure (PAOP) and central venous pressure (CVP) at baseline than the nonresponders. After the fluid challenge, there were no differences between the two groups in MAP, CVP, PAOP or HR. There was no correlation between changes in CI or stroke volume index and baseline SvO_2_. Receiver operating characteristic analysis showed that SvO_2 _was not a predictor of fluid responsiveness.

**Conclusions:**

The response of septic patients to a fluid challenge is independent of baseline SvO_2_. The presence of a high SvO_2 _does not necessarily exclude the need for further fluid administration.

## Introduction

Patients with severe sepsis and septic shock typically have decreased vascular tone, with a high cardiac index (CI), low systemic vascular resistance and elevated mixed venous oxygen saturation (SvO_2_). Fluid resuscitation is essential for the restoration and maintenance of adequate intravascular volume to improve and maintain organ perfusion [1-4]. Natural or artificial colloids or crystalloids may be used for this purpose, as no differences in outcome have been reported related to the type of fluid [5]. As fluid requirements are not easily determined, a fluid challenge technique should be used on a repeated basis according to the patient's response (for example, an increase in blood pressure) and tolerance (for example, excessive increase in cardiac filling pressure) [6-8].

By rearranging the Fick equation, SvO_2 _can be defined as the balance between four variables: CI, hemoglobin, oxygen saturation and oxygen consumption (VO_2_). Monitoring of SvO_2 _therefore allows assessment of total tissue oxygen balance and helps the clinician to determine whether CI and oxygen delivery (DO_2_) are high enough to meet the patient's needs [9,10]. The well-known study by Rivers *et al. *[4] indicated that targeting a mixed central venous oxygen saturation (ScvO_2_) level greater than 70% during early resuscitation of patients with severe sepsis may improve outcomes. However, the measurement of ScvO_2 _or SvO_2 _cannot provide complete information about the reason for the inadequacy between systemic oxygen delivery and demands, and whether these measures can guide therapy in septic patients is unclear. A normal or high SvO_2 _level suggests an adequate CI for tissue energy demands, but may not always indicate adequate fluid resuscitation. Further fluids may be administered in the presence of a normal or high SvO_2 _level to further increase the CI, but whether a fluid challenge is still worth trying when SvO_2 _or ScvO_2 _has reached 70% has not been well defined.

The aim of the present study was to test the hypothesis that preinfusion SvO_2 _values could help predict the response to fluid challenge in critically ill septic patients. We also wanted to define fluid responsiveness in the presence of high SvO_2 _(>70%).

## Materials and methods

In this retrospective study, we reviewed prospectively collected data from patients who had been admitted to a 32-bed university hospital mixed medical-surgical ICU between January 2006 and December 2009. Approval was obtained from the Ethics Committee of Erasme Hospital, and informed consent was waived because of the observational nature of the study. Patients were included if they had met standard criteria for severe sepsis [11], received a fluid challenge during their ICU stay, had a pulmonary artery catheter placed *in situ *and had complete hemodynamic data in our computerized database of hemodynamic profiles. Patients with acute coronary syndrome or a history of cardiac disease were not included, and patients younger than 18 years of age were also excluded.

Disease severity was evaluated by calculation of the Sequential Organ Failure Assessment (SOFA) score [12]. Hemodynamic measurements, arterial oxygen saturation and SvO_2 _values taken before and after fluid challenge were recorded. In our department, fluid challenges are performed when there is a suspicion of hypovolemia based on clinical signs such as oliguria, tachycardia or hypotension. Fluid challenges are conducted according to a standard procedure [8] using 500 mL of colloid or 1,000 mL of crystalloid administered over 30 minutes. Fluids consisted of synthetic colloids (gelatin or pentastarch; Fresenius, Bad Homburg, Germany), albumin 4% (Albumex; CSL, Leuven, Belgium) or crystalloids (Hartmann solution or 0.9% saline solution; Baxter, Lessines, Belgium). The choice of fluid was left to the attending physician. The decision to stop the fluid challenge was based on predetermined safety limits for each patient according to a standard procedure. Respiratory support settings were unchanged during the fluid challenge, and no therapeutic interventions were allowed until new hemodynamic measurements were calculated. Fluid responders were defined as those patients in whom CI increased by at least 10% after fluid challenge.

### Statistical analysis

Statistical analysis was performed using SPSS software (SPSS Inc., Chicago, IL, USA). Student's *t*-test was used for continuous variables. Pearson's correlation was applied. Receiver operating characteristic (ROC) curve analysis was used to assess the predictive ability of fluid responsiveness for SvO_2_, central venous pressure (CVP) and pulmonary artery occlusion pressure (PAOP) values. *P *< 0.05 was considered statistically significant.

## Results

**Figure 1:**
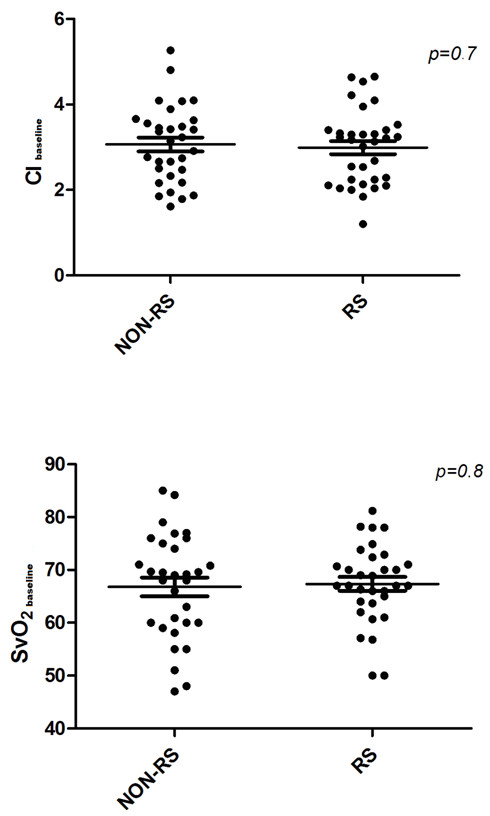
**Scatterplots of baseline CI (top) and SvO_2 _(bottom) values in responders (RS) and nonresponders (NON-RS)**. CI: confidence interval; SvO_2_: mixed venous oxygen saturation. Thin lines represent mean values, and thick lines the standard errors.

Patient characteristics and demographic data are presented in Table 1. Of the 65 patients, 34 (52%) were fluid responders. Among the 34 fluid responders, 17 (50%) had septic shock, and 19 (61%) of the 31 fluid nonresponders had septic shock. The source of sepsis was the respiratory system in 25 patients (38.4%), the abdomen in 16 patients (24.6%), the urinary tract in 8 patients (12.3%) and the bloodstream in 16 patients (24.6%). Sixty of the patients received mechanical ventilation (29 of 31 nonresponders and 31 of 34 responders) in volume-controlled mode (tidal volume 6 to 8 mL/kg and positive end-expiratory pressure (PEEP) 6 to 12 cm H_2_O) or pressure support mode with PEEP 6 to 10 cm H_2_O. Hemodynamic values for fluid responders and nonresponders before and after the fluid challenge are shown in Tables 2 and 3. There were no statistically significant differences between the groups at baseline, except for lower PAOP (*P *= 0.003) and CVP (*P *= 0.008) levels in the responders than in the nonresponders (Figure 1 and Table 2). There were no significant differences in any of the measured variables between the groups after the fluid challenge (Table 2).

**Table 1:** Patients' characteristics and interventions during ICU stay and outcomes^a^

Characteristics	Nonresponders (*n *= 31)	Responders (*n *= 34)	*P *value
Mean age, years	69 ± 14	71 ± 9	0.69
Medical/surgical	22/9	9/25	0.21
Admission SOFA score	10 ± 2	10 ± 4	0.98
Mechanical ventilation	29	31	0.54
Dobutamine/norepinephrine	18/19	16/17	0.58
Hartmann/hetastarch/albumin	19/4/8	23/6/5	0.17
Mortality	18 (58%)	18 (53%)	0.43

^a^SOFA: Sequential Organ Failure Assessment. Data are presented as mean ± SD, *n*, or *n *(%).

**Table 2:** Hemodynamic values in fluid responders and nonresponders before and after fluid challenge^a^

	Before fluid challenge			After fluid challenge		
	
Parameters	Nonresponders (*n *= 31)	Responders (*n *= 34)	*P *value	Nonresponders (*n *= 31)	Responders (*n *= 34)	*P *value
Mean arterial pressure, mmHg	71 ± 8	71 ± 9	0.79	76 ± 8	77 ± 9	0.58
Heart rate, beats/minute	105 ± 21	103 ± 17	0.71	102 ± 21	98 ± 18	0.71
Central venous pressure, mmHg	12 ± 4	9 ± 4	0.008	14 ± 3	12 ± 4	0.18
Pulmonary artery occlusion pressure, mmHg	14 ± 3	12 ± 3	0.003	17 ± 6	14 ± 5	0.18
Mixed venous oxygen saturation, %	67 ± 9	67 ± 7	0.80	67 ± 9	71 ± 6	0.08
Cardiac index, L/minute/m^2^	3 ± 0.9	2.9 ± 0.8	0.73	3.2 ± 0.6	3.6 ± 0.9	0.11

^a^Data are presented as mean ± SD.

**Table 3:** Hemodynamic values before and after fluid challenge in fluid responders and nonresponders^a^

	Nonresponders (*n *= 31)			Responders (*n *= 34)		
	
Parameters	Before	After	*P *value	Before	After	*P *value
Mean arterial pressure, mmHg	71 ± 8	76 ± 8	0.01	71 ± 9	77 ± 9	<0.01
Heart rate, beats/minute	105 ± 21	102 ± 21	0.12	103 ± 17	98 ± 18	0.04
Central venous pressure, mmHg	12 ± 4	14 ± 3	<0.01	9 ± 4	12 ± 4	0.01
Pulmonary artery occlusion pressure, mmHg	14 ± 3	17 ± 6	0.02	12 ± 3	14 ± 5	<0.01
Mixed venous oxygen saturation, %	67 ± 9	67 ± 9	0.97	67 ± 7	71 ± 6	<0.01
Cardiac index, L/minute/m^2^	3 ± 0.9	3.2 ± 0.6	0.23	2.9 ± 0.8	3.6 ± 0.9	<0.01

^a^Data are presented as mean ± SD.

**Figure 2:**
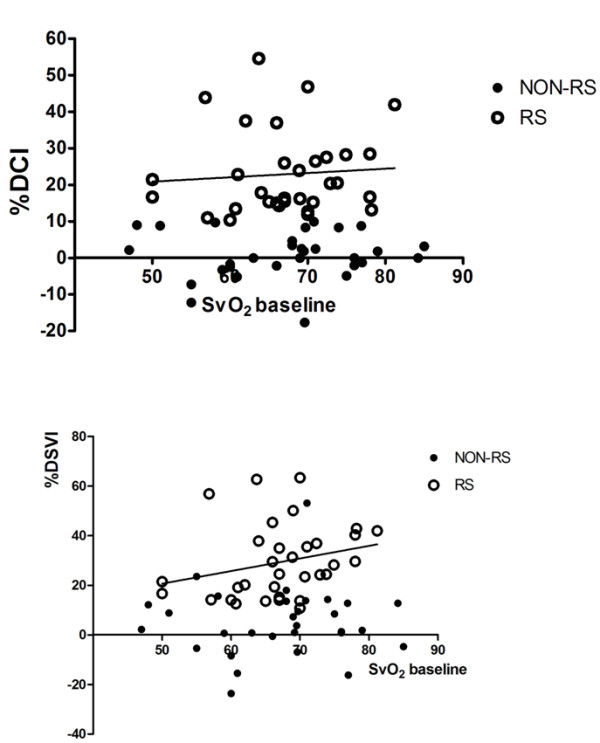
**Relationship between baseline SvO_2 _levels and changes in cardiac index (%DCI) and stroke volume (%DSVI) in all patients (responders [RS] and nonresponders [NON-RS])**. SvO_2_: mixed venous oxygen saturation.

SvO_2 _values in responders and nonresponders are shown in Table 4. There were no differences in baseline lactate levels between patients with higher (>70%) or lower (<70%) SvO_2 _levels (means ± SD: 2.1 ± 1 mg/dL vs. 2.6 mg/dL ± 1.9, respectively; *P *= 0.279). There were no correlations between change in CI (%DCI) or change in stroke volume index (DSVI%) and baseline SvO_2 _in all patients (Figure 2). In the subgroup of patients with high baseline SvO_2 _levels (>70%), there was also no correlation between %DCI and SvO_2 _levels. Similar findings were observed in the subgroup of patients with lower SvO_2 _values (<70%).

**Table 4:** Numbers of fluid responders and nonresponders in different ranges of SvO_2 _values^a^

SvO_2_	Nonresponders (*n *= 31)	Responders (*n *= 34)	Proportion of responders
<50%	3	2	40%
50% to 60%	8	4	33%
60% to 70%	8	15	65%
>70%	12	13	52%

^a^SvO_2_: mixed venous oxygen saturation.

**Figure 3:**
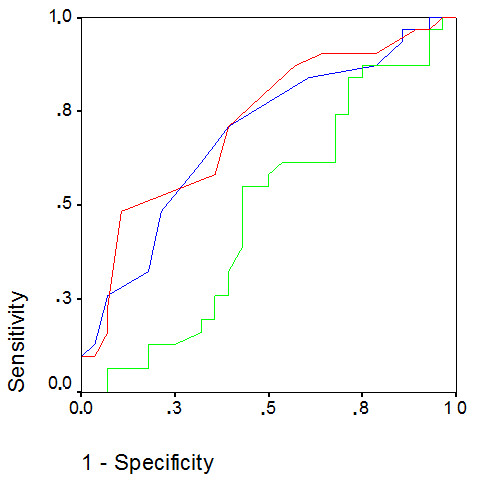
**ROC curves for baseline values of SvO_2 _(green line), CVP (blue line) and PAOP (red line)**. Diagonal segments are produced by ties. AUC were 0.51, 0.68 and 0.71 for SvO_2_, CVP and PAOP, respectively. CVP at a cutoff of 11 mmHg had a sensitivity of 67% and a specificity of 54% for predicting fluid responsiveness. PAOP at a cutoff of 13 mmHg had a sensitivity of 59% and a specificity of 65%. AUC: area under the curve; CVP: central venous pressure; PAOP: pulmonary artery occlusion pressure; ROC: receiver operating characteristic; SvO_2_: mixed venous oxygen saturation.

ROC curves showed that baseline SvO_2 _level was not a good predictor of %DCI after fluid challenge (area under the curve (AUC) = 0.51, 95% confidence interval = 0.36 to 0.66; *P *= 0.85). Baseline CVP was an adequate predictor of %DCI (AUC = 0.68, 95% confidence interval = 0.54 to 0.83; *P *= 0.01), but with an unsatisfactory sensitivity and specificity (67% and 54%, respectively). Similar findings were noted for PAOP (AUC = 0.71, 95% confidence interval = 0.58 to 0.86; *P *= 0.04) (sensitivity and specificity, 59% and 65%, respectively) (Figure 3).

## Discussion

The main finding of our study is that the response to fluid challenge was independent of baseline SvO_2 _levels, with no correlation between baseline SvO_2 _levels and %DCI or DSVI% after fluid challenge. This is an important new finding. Moreover, high SvO_2 _levels did not correlate with a specific response to fluid challenge, as 13 (52%) of the 25 patients with SvO_2 _>70% responded to fluid challenge. SvO_2 _levels do not, therefore, predict responsiveness to fluid challenge in patients with severe sepsis.

The treatment of patients with severe sepsis and septic shock aims to restore and maintain hemodynamic parameters at levels that are able to sustain tissue and cellular perfusion. Fluid resuscitation plays a key role in this therapeutic strategy, and hemodynamic monitoring plays an important role in optimizing management. Low SvO_2 _is often associated with an inadequate CI, low arterial oxygen content or increased VO_2 _by the tissues and thus may suggest the need for an intervention to increase DO_2 _to the tissues. A main target in the early goal-directed therapy protocol in septic patients proposed by Rivers and co-workers [4] is to maintain SvO_2 _at least 70% and not to target a specific CI value. However, the patients in their study were in the emergency department before fluid resuscitation, and these patients had unexpectedly low ScvO_2 _values. ScvO_2 _is a measure of the oxygenation in blood coming from the upper body and cannot provide information about the global inadequacy between systemic oxygen delivery and tissue oxygen demands in such patients. As the pulmonary artery carries blood from all vascular beds, SvO_2 _better reflects the amount of oxygen left after passage through the tissues. However, evaluation of SvO_2 _may not provide a correct assessment of tissue oxygenation, particularly in the setting of septic shock, when oxygen extraction is altered. Nevertheless, SvO_2 _values must be interpreted within the context of the overall hemodynamic profile in septic patients [5,13]. SvO_2 _is still a global parameter and gives no specific information on regional tissue oxygenation, but neither does CI [14]. Importantly, a low SvO_2 _value is a warning sign of potential inadequacy of oxygen delivery for tissue demands, suggesting the need to increase oxygen delivery to the tissues with further fluids or transfusions or dobutamine administration. However, a normal or high SvO_2 _level does not necessarily indicate that oxygen metabolism is entirely normalized and does not exclude the presence of persisting tissue hypoxia. Microcirculatory shunting in sepsis can result in a normal SvO_2 _level despite local tissue dysoxia, and patients can develop multiple organ failure and die with supranormal SvO_2 _values [14].

Conversely, several patients may have responded to fluid administration, even though they had an adequate CI. Fluid responsiveness does not necessarily mean that fluid administration is mandatory. We cannot verify whether the increase in CI in the patients with a high SvO_2 _was associated with improved outcomes, as this was not the aim of the study. In our study, we chose a cutoff SvO_2 _value of 70% as suggested by Pinsky and Vincent [15], because our patients were at the late stage of sepsis, and not 65% as proposed by the Surviving Sepsis Campaign Guidelines, which referred to the early resuscitation phase [5]. Nevertheless, it is not clear which value should be considered as normal for septic patients in the ICU. In sepsis, DO_2_-VO_2 _relationships are altered and arterioventricular shunting may increase SvO_2_. Correction of hypovolemia with fluids and restoration of the distributed microcirculation is necessary, but SvO_2 _alone seems to be an inadequate parameter as a guide for therapy. Gattinoni *et al. *[16], in a study of surgical patients admitted to the ICU after developing organ failure, failed to show improved outcomes with therapies aimed at maintaining either DO_2 _or SvO_2 _at supranormal values. In a meta-analysis, Heyland *et al. *[17] demonstrated that therapy targeted at supraphysiologic end points (DO_2 _and VO_2_) was not associated with decreased mortality. Although it is impossible to establish an absolute "normal" value of SvO_2_, in most clinical situations, SvO_2 _levels ranging from 60% to 70% suggest that tissue DO_2 _is adequate. Usually, changes in SvO_2 _are more informative than absolute SvO_2 _values. The results of our study suggest that SvO_2 _and, in particular, high SvO_2 _levels cannot serve as predictors of fluid responsiveness in critically ill septic patients, as 52% of septic patients with SvO_2 _>70% responded to fluids by increasing their CI values >10%.

There was no significant difference in other baseline variables between responders and nonresponders, except for CVP and PAOP, which were lower in the responders. Furthermore, the ROC analysis showed poor sensitivity and specificity for CVP and PAOP, indicating that targeting volume therapy to these filling pressure values should be discouraged. Our results are in agreement with those found in other clinical studies that showed that CVP and PAOP were not reliable predictors of volume responsiveness in sepsis [3,18-20]. Some authors have demonstrated that filling pressures have a low predictive value for estimating fluid responsiveness during mechanical ventilation in septic patients and suggested that using them to guide fluid therapy can lead to inappropriate therapeutic decisions [21,22]. In a recent systematic review of the literature, researchers found a lack of agreement on hemodynamic goals for the management of sepsis and proposed that this lack of consistency may contribute to heterogeneity in treatment effects in clinical trials of novel sepsis therapies [23]. It is likely that many variables need to be assessed together at the bedside to enable the most effective treatment of patients.

The present study has some limitations. First, we do not have information on the exact time of onset of severe sepsis; it would have been interesting to differentiate patients with early and late sepsis. Furthermore, this retrospective, observational study was conducted with a relatively small sample size and a highly selected cohort in that only patients who required a pulmonary artery catheter were included. Nevertheless, it represents a useful pilot study for further prospective investigations with larger numbers of patients.

## Conclusions

Although SvO_2 _monitoring has been shown to be a useful tool to evaluate the balance between oxygen consumption and supply, SvO_2 _levels before fluid challenge are poor predictors of fluid responsiveness in patients with severe sepsis. The presence of a normal or high SvO_2 _does not necessarily exclude the need for further fluid administration.

## Key messages

• Evaluation of SvO_2 _in patients with sepsis cannot provide information about the reason for the inadequacy between oxygen delivery and demand.

• The presence of a normal or high SvO_2 _level does not necessarily indicate that oxygen metabolism is normalized.

• The presence of a normal or high SvO_2 _level does not exclude the need for the administration of further fluids.

• SvO_2 _levels are poor indicators of fluid responsiveness in septic patients.

## Abbreviations

AUC: area under the curve; CI: cardiac index; CVP: central venous pressure; DO_2_: oxygen delivery; HR: heart rate; MAP: mean arterial pressure; PAOP: pulmonary artery occluded pressure; PEEP: positive end-expiratory pressure; ROC: receiver operating characteristic; ScvO_2_: central venous oxygen saturation; SOFA: Sequential Organ Failure Assessment; SVI: stroke volume index; SvO_2_: mixed venous oxygen saturation; VO_2_: oxygen uptake.

## Competing interests

The authors declare that they have no competing interests.

## Authors' contributions

DV participated in the design of the study, collected the data, performed the statistical analysis and drafted the manuscript. CP and SS helped collect the data. DDB and JLV participated in the design of the study and helped revise the manuscript. All authors read and approved the final manuscript.
